# Functional Characterization of Target of Rapamycin Signaling in *Verticillium dahliae*

**DOI:** 10.3389/fmicb.2019.00501

**Published:** 2019-03-13

**Authors:** Linxuan Li, Tingting Zhu, Yun Song, Xiumei Luo, Li Feng, Fengping Zhuo, Fuguang Li, Maozhi Ren

**Affiliations:** ^1^School of Life Sciences, Chongqing University, Chongqing, China; ^2^Zhengzhou Research Base, State Key Laboratory of Cotton Biology, Zhengzhou University, Zhengzhou, China; ^3^National Key Laboratory of Cotton Biology, Institute of Cotton Research, Chinese Academy of Agricultural Sciences, Anyang, China; ^4^School of Chemistry and Chemical Engineering, Chongqing University of Science and Technology, Chongqing, China

**Keywords:** rapamycin, target of rapamycin, pathogenicity, *Verticillium dahliae*, Verticillium wilt

## Abstract

More than 200 plants have been suffering from Verticillium wilt caused by *Verticillium dahliae* (*V. dahliae*) across the world. The target of rapamycin (TOR) is a lethal gene and controls cell growth and development in various eukaryotes, but little is known about TOR signaling in *V. dahliae*. Here, we found that *V. dahliae* strain is hypersensitive to rapamycin in the presence of rapamycin binding protein VdFKBP12 while the deletion mutant aaa*vdfkbp12* is insensitive to rapamycin. Heterologous expressing *VdFKBP12* in *Arabidopsis* conferred rapamycin sensitivity, indicating that *VdFKBP12* can bridge the interaction between rapamycin and TOR across species. The key across species of TOR complex 1 (TORC1) and TORC2 have been identified in *V. dahliae*, suggesting that TOR signaling pathway is evolutionarily conserved in eukaryotic species. Furthermore, the RNA-seq analysis showed that ribosomal biogenesis, RNA polymerase II transcription factors and many metabolic processes were significantly suppressed in rapamycin treated cells of *V. dahliae*. Importantly, transcript levels of genes associated with cell wall degrading enzymes (CWEDs) were dramatically down-regulated in TOR-inhibited cells. Further infection assay showed that the pathogenicity of *V. dahliae* and occurrence of Verticillium wilt can be blocked in the presence of rapamycin. These observations suggested that VdTOR is a key target of *V. dahliae* for controlling and preventing Verticillium wilt in plants.

## Introduction

*Verticillium dahliae* (*V. dahliae*) is a soil-borne and hemibiotrophic fungus that causes over 200 plant species wilting including *Brassicaceae*, *Rosaceae*, and *Solanaceae* plants, resulting in tremendous economic losses every year (Pegg and Brady, 2002; Inderbitzin and Subbarao, 2014; Gomez-Lama Cabanas et al., 2015; Chen et al., 2017). *V. dahliae* is the agent of Verticillium wilt, which is one of the most devastating cotton diseases worldwide. *V. dahliae* is particularly difficult to manage because it long exists in soil as a dormant structure called microsclerotia. The microsclerotia are primary infectious propagules that can remain alive in soil for more than 20 years (Agrios, 2005; Bui et al., 2018). Hyphopodium differentiates from hypha after conidia germination on the root surface and develops a penetration peg to infect plant roots (Zhao et al., 2016). Hyphal neck from penetration peg partitions the hyphopodium and the invasive hypha and forms a specialized fungus–host interface to deliver secretory proteins into host (Zhou et al., 2017). The plant cell wall is an important interface for the interaction between host and phytopathogenic fungi, which plays a major barrier role in the process of phytopathogenic fungi invading the host. Most fungal pathogens secrete lots of cell wall degrading enzymes (CWDEs) including cellulases, xylanases, and pectinases to depolymerize the host cell wall (Tonukari, 2003; Quoc and Chau, 2017). *V. dahliae* have been reported to produce CWDEs for degrading plant cell wall (Cooper and Wood, 1980; Tzima et al., 2011; Chen et al., 2016). Endoglucanase-1 (EG-1) is an important enzyme in depolymerization of plant cellulose (Novo et al., 2006; Valaskova and Baldrian, 2006). The *EG-1* gene homolog *VdEg-1* plays an important role in plant penetration and colonization. The *VdEg-1* mutant lost the ability to colonize vascular tissues in inoculated plants (Maruthachalam et al., 2011). Moreover, pectinases play a critical role in pathogenesis and production levels correlated with pathogenicity in different *Verticillium* strains (Durrands and Cooper, 1988; Fradin and Thomma, 2006; Tzima et al., 2011; Chen et al., 2016).

Target of rapamycin (TOR) is an evolutionarily conserved phosphoinositide-3 kinase-related protein kinase that controls multiple cellular processes in response to various intracellular and extracellular signals (De Virgilio and Loewith, 2006; Shimobayashi and Hall, 2014; Dobrenel et al., 2016; Saxton and Sabatini, 2017). It was originally identified in budding yeast through mutant screens for resistance to the immunosuppressant drug rapamycin (Heitman et al., 1991a). Subsequent identification of TOR in humans and other eukaryotes revealed evolutionary conservation of TOR from the last eukaryotic common ancestor to humans (Soulard et al., 2009; Katz, 2012; Tatebe and Shiozaki, 2017). TOR exists in two functionally and structurally distinct complexes: TOR complex 1 (TORC1) and TORC2. The essential core components of TORC1 are TOR, RAPTOR (regulatory-associated protein of TOR) and LST8 (lethal with SEC thirteen 8), which controls cell growth by regulating translation, transcription and autophagy (Wang and Proud, 2009; Iadevaia et al., 2014; Dobrenel et al., 2016); whereas, those of TORC2 are TOR, RICTOR (rapamycin-insensitive companion of TOR), SIN1 (SAPK-interacting 1) and LST8 (Hara et al., 2002; Jacinto et al., 2004; De Virgilio and Loewith, 2006; Gaubitz et al., 2016). TORC2 responds primarily to growth factors, promoting cell survival, cell cycle and actin cytoskeleton polarization (Jacinto et al., 2004; Oh and Jacinto, 2011; Gaubitz et al., 2016).

Rapamycin (RAP) is a new macrolide immunosuppressant drug produced by *Streptomyces hygroscopicus*. RAP specifically binds to FKBP12 (FK506 binding protein of 12 kD), which interacts with the FRB domain of TOR kinase to inhibit TORC1 activity (Heitman et al., 1991b; Hara et al., 2002). Crystal structure showed that the ternary complex of RAP-FKBP12-FRB domain of TOR partially occludes substrates to the active site of TOR (Yang et al., 2013; Aylett et al., 2016). However, TORC2 is RAP insensitive (Loewith et al., 2002). The C-terminus of RICTOR prevents the RAP-FKBP12 complex from binding to the FRB domain of TOR protein in TORC2, which makes TORC2 insensitive to RAP (Gaubitz et al., 2015). In addition, the ATP-competitive TOR protein kinase inhibitors including Torin1, Torin2, Ku-0063794 and AZD-8055 can directly bind to the kinase domain of TOR by competing with ATP to inhibit TORC1 and TORC2 activity (Garcia-Martinez et al., 2009; Chresta et al., 2010; Liu et al., 2010, 2011).

The TOR signaling pathway is a central regulator in regulating cell growth, proliferation and metabolism from yeasts to humans (Rexin et al., 2015; Dobrenel et al., 2016; Saxton and Sabatini, 2017). There is no research on the function of TOR signaling pathway in *V. dahliae*. In this study, we found that the mycelial growth of *V. dahliae* was retarded by RAP, implying that VdFKBP12 may be functional in mediate RAP and VdTOR. Further functional analysis of aaa*vdfkbp12* and *VdFKBP12* overexpression transgenic *Arabidopsis* suggests that VdFKBP12 can mediate the inhibition of TOR kinase by RAP in *V. dahliae*. The conserved TOR signaling pathway including TORC1 and TORC2 existing in *V. dahliae*, indicating that TOR signaling pathway is evolutionarily conserved in eukaryotic species. Additionally, RNA-seq experiments were performed to test the function of VdTOR. A large number of differentially expression genes (DEGs) involving in various cellular processes, such as ribosome biogenesis and CWDEs, were observed in RAP treatment. Importantly, the most of CWDEs are down-regulated in TOR-inhibited cells, implying that TOR involved in the regulation of invasion. Infection assay showed that the pathogenicity of *V. dahliae* and occurrence of Verticillium wilt can be blocked in the presence of RAP. These independent evidences indicated that RAP inhibits mycelial growth and pathogenicity through reducing VdTOR activity in *V. dahliae*.

## Materials and Methods

### Fungal Strains and Culture Conditions

The highly aggressive defoliating isolate Vd991 of *V. dahliae* was used as the wild-type (WT) strain in this study. The WT strain, deletion mutants and complemented strains were cultured on potato dextrose agar (PDA) at 27°C. For extraction of genomic DNA and conidia production, hyphae were incubated in potato dextrose broth (PDB) at 27°C with shaking at 160 rpm.

### Construction of Vectors for Gene Deletion and Complementation

The primers for gene deletion and complementation were listed in Supplementary Table 1. Constructs for gene deletion and complementation of *V. dahliae* were carried out as described previously (Luo et al., 2016). *Agrobacterium tumefaciens* strain AGL-1 was used to transform the conidia of *V. dahliae* by using ATMT. The *Agrobacterium tumefaciens* strain AGL-1 containing gene deletion or complementation vector was mixing with equal volume of conidial suspension of *V. dahliae* (10^7^ conidia. mL^-1^). Then, 200 μL of the mixture was placed onto microporous membranes (pore size, 0.45 μm) on cocultivation medium for 48 h. Subsequently, the membranes were transferred to PDA medium containing hygromycin antibiotic (50 mg. mL^-1^) and cefotaxime (300 mg. mL^-1^). After 10 days, transformants were transferred to fresh PDA medium with hygromycin (50 mg. mL^-1^) for further analysis.

### Expression Profiling Sequencing and Analysis

Spores of *V. dahliae* were grown for 5 days in PDB medium at 27°C with shaking at 160 rpm, and then treated with 5 nM RAP and DMSO (as a control) for 24 h, respectively. Total RNA of *V. dahliae* mycelium was isolated using the Hipure Fungal RNA Kit (Magen, Guangzhou, China). For each treatment, three independent biological replicates were performed. The cDNA library construction was done as described previously (Marioni et al., 2008). An Illumina Hiseq 2000 platform was used to sequence these libraries. The clean reads were mapped to the reference *V. dahliae* genome database website^1^ by using TopHat2 software (Kim et al., 2013). Cufflinks and Cuffdiff were used to assemble the mapped reads and identify differentially expressed genes (DEGs), respectively (Trapnell et al., 2010, 2013). Gene ontology (GO) enrichment (corrected *P*-value < 0.05) of DEGs was performed by using GOseq R package software (Young et al., 2010). The enrichment of DEGs in Kyoto Encyclopedia of Genes and Genomes (KEGG) pathways (corrected *P*-value < 0.05) was obtained by using KOBAS software (Kanehisa et al., 2008).

### Quantitative Real-Time PCR

Total RNA of *V. dahliae* which treated with DMSO and RAP (5 nM) for 24 h in PDB medium was extracted using the Hipure Fungal RNA Kit (Magen, Guangzhou, China). The software Primer premier 5.0 was used to design quantitative real-time primers (Supplementary Table 2). *Vd18S rRNA* was used as a control. Reaction was performed in a final volume of 25 μL containing 12.5 μL of 2 × SYBR^®^Premix Ex Taq (Takara, Dalian, China). The relative expression level of each target gene was analyzed with the Bio-Rad CFX Manager software. The data represented the mean ± SD of three independent experiments.

### Pathogen Inoculation and Cellophane Penetration Assays

Pathogen inoculation was performed by root dip inoculation with conidia of *V. dahliae* (10^7^ conidia. mL^-1^) as described previously (Luo et al., 2016). Plants of similar height were selected for each treatment. For the root dip inoculation, 5-week-old roots of cotton plants were immersed in 200 mL of conidia suspension supplemented with or without 50 nM RAP for 10 min. Equal volume DMSO as a solvent control, the final concentration of DMSO was 0.25% (v/v). Then the plants were re-cultivated in soil until the disease symptoms appeared. The roots of cotton plants were immersed in 200 mL sterile water for 10 min and then re-cultured in soil as a control. Cellophane penetration assay was performed as described previously (Prados Rosales and Di Pietro, 2008). The experiments were repeated at least three times.

### Measurement of Cellulose and Pectin Content

Cellulose content was measured with the anthrone method previously described (Ververis et al., 2004). Cellulose content of the sample was measured by ultraviolet spectrophotometer of absorbance at 620 nm. Commercial cellulose was used as control for standard curve.

Pectin content was calculated based on the analysis of uronic acid content. The content of uronic acids was measured with the biphenol method as described previously (Blumenkrantz and Asboe-Hansen, 1973). The content of uronic acids was measured by ultraviolet spectrophotometer of absorbance at 525 nm. Commercial galacturonic acid was used as a standard for the calibration curve.

### Combination Index (CI) Value Measurement

Combination index (CI) values were used to evaluate the interaction between RAP and Torin1. The degree of reagents interaction is based on synergistic effect (CI < 1), additive effect (CI = 1), or antagonism (CI > 1) (Chou, 2006). Spores of *V. dahliae* were treated with different concentrations RAP and Torin1, or pairwise combination of RAP + Torin1 on PDA medium for 11 days at 27°C. Colony diameter was measured to calculate CI values. Experiments were repeated at least three times. The values of affected fraction (Fa) were calculated according to the CompuSyn sofware program (Chou and Talalay, 1984).

## Results

### RAP Can Inhibit the Mycelial Growth and Conidial Development of *V. dahliae* in a Dose Dependent Manner

**FIGURE 1:**
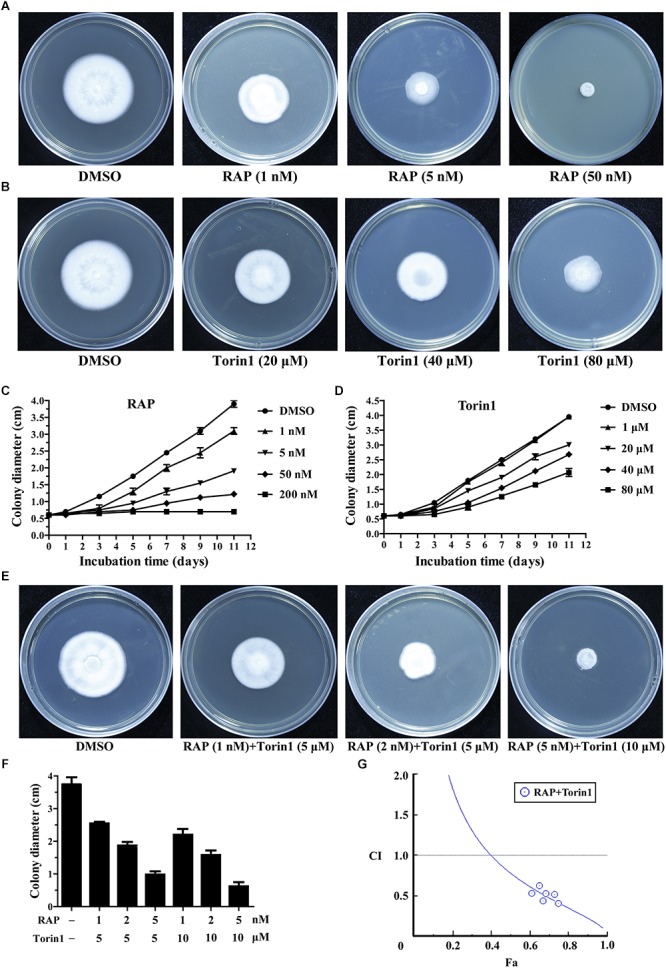
*V. dahliae* was sensitive to RAP and Torin1 in a dose dependent manner. **(A)** Spores of *V. dahliae* were incubated on potato dextrose agar (PDA) including different concentrations RAP for 11 days. **(B)** Spores of *V. dahliae* were incubated on PDA medium including different concentrations Torin1 for 11 days. **(C)** Colony diameter of *V. dahliae* was incubated on PDA medium including different concentrations RAP from 0 to 11 days. The data represents the mean ± SD of *n* = 3 independent experiments. **(D)** Colony diameter of *V. dahliae* was incubated on PDA medium including different concentrations Torin1 from 0 to 11 days. The data represents the mean ± SD of *n* = 3 independent experiments. **(E)** Spores of *V. dahliae* were incubated on PDA including different concentrations combination of RAP and Torin1 for 11 days. **(F)** Colony diameter of *V. dahliae* was incubated on PDA medium including different combination of RAP and Torin1 for 11 days. The data represents the mean ± SD of *n* = 3 independent experiments. **(G)** Fa-CI curve shows synergism (CI < 1) between RAP and Torin1.

**FIGURE 2:**
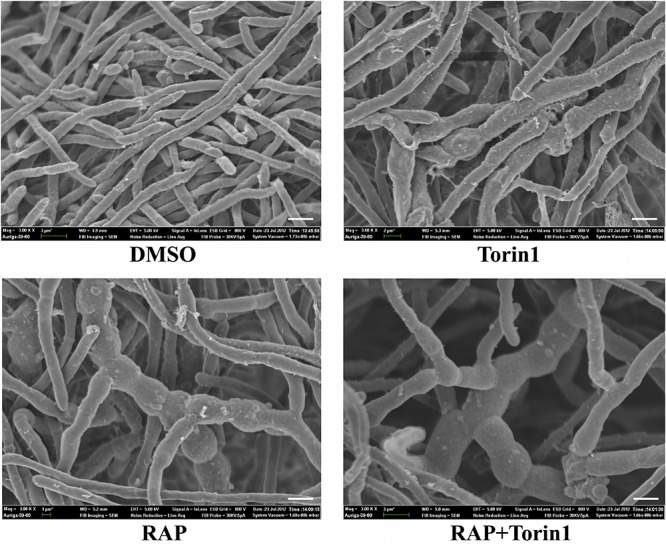
TOR inhibitors RAP and Torin1 can inhibit the mycelial growth of *V. dahliae.* Hyphae were incubated in potato dextrose broth (PDB) with RAP (5 nM) and Torin1 (80 μM) for 24 h, and then were photographed by scanning electron microscopy. Bar = 3 μm.

*V. dahliae* is the main pathogen of Verticillium wilt, which is a devastating plant disease that causes a variety of economic crops wilting, including cotton, tomato, and eggplant (Pegg and Brady, 2002; Inderbitzin and Subbarao, 2014). Rapamycin (RAP) is a broad-spectrum antifungal drug that effectively inhibits pathogenic fungi (Bastidas et al., 2008; Benjamin et al., 2011; Yu et al., 2014). In order to test the antifungal activity of RAP on *V. dahliae*, RAP was applied to *V. dahliae*. As expected, with increasing concentrations of RAP, the hyphal growth was subjected to different degrees of inhibition (Figure 1A,C). Meanwhile, Torin1, the second-generation inhibitor of TOR, also inhibits hyphal growth in a dose dependent manner (Figure 1B,D). The IC50 (half-maximal inhibitory concentration) values of RAP and Torin1 were 5 nM and 80 μM, respectively. These results indicated that RAP has a stronger inhibitory effect than Torin1. This may be due to the specific spatial structure of VdTOR, which requires a high concentration of Torin1 to inhibit the kinase activity of VdTOR. Furthermore, the pairwise combination of RAP and Torin1 had enhanced inhibition for hyphal growth compared with RAP or Torin1 alone treatment. The single IC50 value (RAP: 2 nM; Torin1: 5 μM) was significantly reduced when *V. dahliae* was subjected to RAP and Torin1 combination treatment (Figure 1E,F). The results implied that the potential synergistic effects can be generated by combining RAP and Torin1. Next, Fa-CI curve was generated by using the CompuSyn software (Chou, 2006). Synergistic effect (CI < 1) was observed when hyphae were treated with combination of RAP and Torin1 (Figure 1G), showing that the combination of RAP and Torin1 may have synergistic effect. Additionally, swollen hyphae and shorter septation were observed in RAP, Torin1, and RAP + Torin1 treatment compared with the control (DMSO) (Figure 2). These results implied that RAP and Torin1 might synergistically inhibit hyphal growth in *V. dahliae*.

To determine whether RAP and Torin1 inhibit conidial development, we examined germination rate of conidia and spore production. The germination rate of conidia and spore production were obvious reduction by RAP and Torin1 treatment (Supplementary Figure 1A). Meanwhile, the expression levels of genes involved in conidial development including *VDAG_JR2_Chr4g03070* (*Vdcon10*), *VDAG_JR2_Chr2g09020* (*Vdfluffy*), *VDAG_JR2_Chr7g08730* (*VdPKAC1*) and *VDAG_JR2_Chr8g02550* (*VdSge1*) genes (Tzima et al., 2010; Santhanam and Thomma, 2013) were significantly down-regulated in RAP and Torin1 treatment (Supplementary Figure 1B). These data suggested that RAP and Torin1 can inhibit conidial development in *V. dahliae*. Besides, we examined the expression levels of genes involved in vegetative growth and virulence (Klimes and Dobinson, 2006; Gao et al., 2010; Zhou et al., 2012). The expression of *VDAG_JR2_Chr2g02500* (*VDH1*), *VDAG_JR2_Chr3g07080* (*GARP1*), *VDAG_JR2_Chr6g08770* (*NLP1*), and *VDAG_JR2_Chr2g05460* (*NLP2*) were also significantly down-regulated in RAP and Torin1 treatment (Supplementary Figure 1C), suggesting that TOR inhibition reduced expression levels of vegetative growth and virulence-related genes.

### The Conserved TOR Signaling Pathway Existing in *V. dahliae*

**FIGURE 3:**
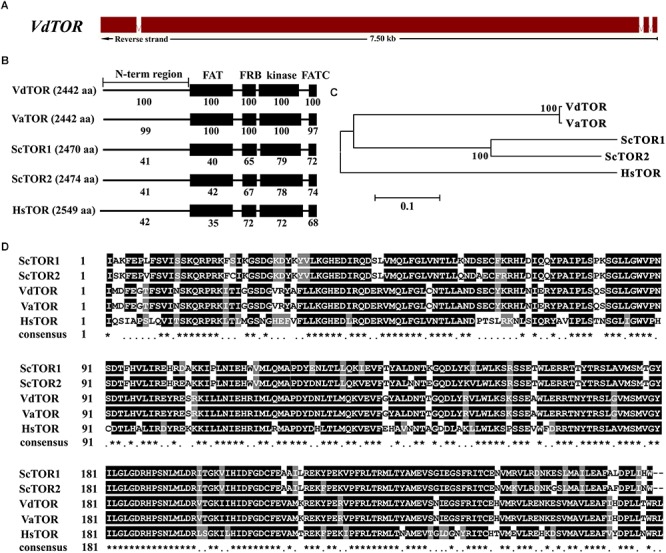
Sequence and structure analysis of *VdTOR* gene. **(A)** Sequence of *VdTOR* gene. Red represents exons and the white rectangles signify introns. **(B)** Comparison conserved domains of VdTOR proteins with that from other organisms. Each value indicates the percentage of identity with the corresponding domain sequences of VdTOR. The number in brackets represents the number of amino acids. Vd, *Verticillium dahliae*; Va, *Verticillium alfalfa*; Hs, *Homo sapiens*; Sc, *Saccharomyces cerevisiae*. **(C)** Phylogenetic relationship between the VdTOR protein and homologs from other organisms. The phylogenetic tree was generated with MEGA4.0 using the neighbor-joining methods. **(D)** Comparison of amino acid sequences of the kinase domain of VdTOR protein with that from other representative organisms. The ^∗^ represents identical amino acid residues.

Rapamycin is a well-known TOR inhibitor which specifically targets the TOR protein (Benjamin et al., 2011). TOR is a central regulator of cell growth and metabolism in various eukaryotic species from yeasts, plants to humans (Yu et al., 2014; Rexin et al., 2015; Saxton and Sabatini, 2017). In order to identify evolutionary conserved TOR signaling pathway components in *V. dahliae*. A BLASTp analysis of the *V. dahliae* genome database (see footnote 1) was performed by using yeast TOR signaling pathway components as reference. A putative homologous gene encoding the key TOR protein (*VDAG_JR2_Chr6g10810*) locates on chromosome 6 was found in *V. dahliae* genome database (Table 1). The *TOR* gene sequence contains 3 introns and 4 exons, which encodes 2442 amino acid residues with molecular mass of 276 kDa (Figure 3A). Alignment of VdTOR with other species TOR proteins showed similar conserved domains including N-term region, FAT, FRB, kinase, and FATC domains as yeast (Figure 3B). Phylogenetic analysis (Figure 3C) and kinase domain alignment with that from other organisms (Figure 3D) indicated that VdTOR was evolutionarily conserved. To further determine VdTOR function, targeted gene replacement was performed in *V. dahliae* strain. All of hygromycin-resistant transformants of *VdTOR* deletion mutants were ectopic mutants and failure in creating a null mutant, implying that deletion of *VdTOR* gene may be lethal. Besides, we also found other homologous genes encoding the key proteins of TORC1 including RAPTOR and LST8. Meanwhile, putative homologs of TORC2 specific proteins including RICTOR and SIN1 were also present in *V. dahliae* genome (Table 1). These results indicated that exist a conserved and functional TORC1 and TORC2 in *V. dahliae*.

**Table 1:** The putative components of TOR signaling pathway in *Verticillium dahliae*.

Protein name	*Homo sapiens*	Yeast	*Verticillium dahliae*	Identity (%)	Chr
Target of rapamycin	mTOR	TOR1	VdTOR-like VDAG_JR2_Chr6g10810a	46	6
		TOR2			
Regulatory associate protein of TOR	mRAPTOR	KOG1	VdRAPTOR-like VDAG_JR2_Chr2g07540a	36	2
Lethal with sec-13 protein 8	mLST8	LST8	VdLST8-like VDAG_JR2_Chr1g16920a	64	1
FK506-binding protein 12	mFKBP12	FPR1	VdFKBP12-like VDAG_JR2_Chr1g18420a	57	1
Stress activated map kinase-interacting protein 1	mSIN1	AVO1	VdSIN1-like VDAG_JR2_Chr7g08790a	30	7
Adhere voraciously to TOR2	/	AVO2	VdAVO2-like VDAG_JR2_Chr7g08290a	30	7
Rapamycin-insensitive companion of mTOR	mRICTOR	AVO3	VdRICTOR-like VDAG_JR2_Chr5g09570a	27	5
Type 2A phosphatase associated protein 42	mIGBP1	TAP42	VdIGBP1-like VDAG_JR2_Chr1g18890a	29	1
S6 kinase	mS6K	SCH9	VdS6K-like VDAG_JR2_Chr3g02040a	55	3
Sfp1	/	Sfp1	VdSfp1-like VDAG_JR2_Chr7g01990a	25	7
Ribosome protein small subunit 6	mRPS6	RPS6A	/	/	/
		RPS6B	VdRPS6B-like VDAG_JR2_Chr4g06860a	61	4
eIF2α kinase	mEIF2AK	GCN2	VdEIF2AK-like VDAG_JR2_Chr1g11510a	31	1
Serine/threonine MAP kinase	MAPK	MPK1	VdMAPK-like1 VDAG_JR2_Chr1g25580a	69	1
			VdMAPK-like2 VDAG_JR2_Chr2g01260a	54	2
AMP activated protein kinase	mAMPK	SNF1	VdAMPK-like VDAG_JR2_Chr1g13020a	40	1
Catalytic subunit of protein phosphatase 2A	mPP2CA	PPH21	VdPP2CA-like VDAG_JR2_Chr1g19620a	78	1
Serine/threonine-protein phosphatase PP1-1	mPP6C	SIT4	VdPP6C-like VDAG_JR2_Chr2g05180a	73	2
Type 2A phosphatase activator TIP41	mTIPRL	TIP41	VdTIPRL-like VDAG_JR2_Chr7g08900a	38	7
Eukaryotic translation initiation factor 2 subunit alpha	meIF2α	eIF2α	VdeIF2α-like VDAG_JR2_Chr2g06100a	65	2


### RAP Inhibited the Activity of VdTOR Protein by VdFKBP12 in *V. dahliae*

**FIGURE 4:**
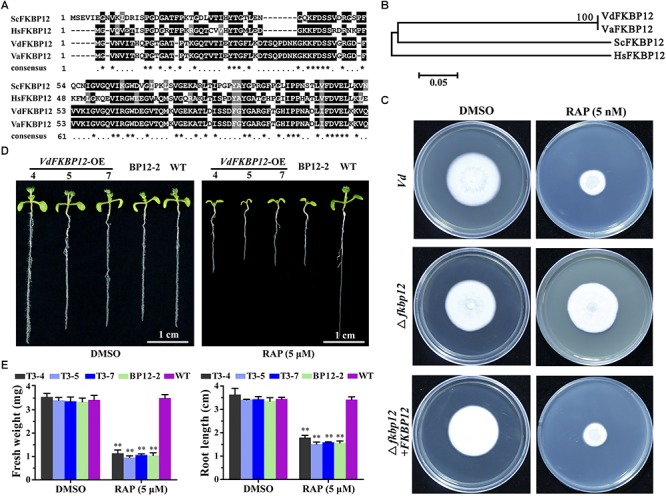
Rapamycin binds to FKBP12 to inhibit TOR activity in *V. dahliae*. **(A)** Comparison of amino acid sequences of the FKBP12 from Vd, Va, Hs and Sc. The ^∗^ represents identical amino acid residues. Vd, *Verticillium dahliae*; Va, *Verticillium alfalfa*; Hs, *Homo sapiens*; Sc, *Saccharomyces cerevisiae*. **(B)** Phylogenetic relationship between *V. dahliae* FKBP12 protein and homologs from other organisms in **(A)**. The phylogenetic tree was generated with MEGA4.0 using the neighbor-joining methods. **(C)** Deletion of *VdFKBP12* (aaa*fkbp12*) leads to resistance to RAP in *V. dahliae*. Spores of *Vd*, aaa*fkbp12* and the complementary strain (aaa*fkbp12*+*FKBP12*) were incubated on PDA including 5 nM RAP for 11 days. **(D)** VdFKBP12 overexpression transgenic *Arabidopsis* lines were sensitive to RAP (5 μM) treatment. Bar = 1 cm. **(E)** Fresh weight and root length of *VdFKBP12* overexpression transgenic *Arabidopsis* lines treated with RAP (5 μM). The data represents the mean ± SD of *n* = 3 independent experiments. Asterisks denote Student’s *t*-test significant difference compared with WT plants (^∗∗^*P* < 0.01).

Rapamycin specifically forms non-covalent to link the interaction between FKBP12 and FRB domain of TOR protein. The formation of the ternary complex of RAP-FKBP12-FRB domain of TOR is necessary for RAP response in eukaryotes (Menand et al., 2002; Sormani et al., 2007). *V. dahliae* owns one *FKBP12* ortholog *VdFKBP12* (*VDAG_JR2_Chr1g18420*) locating on chromosome 1, which encodes a protein with 57% similarity to ScFKBP12 (Table 1). The amino acid sequences alignment (Figure 4A) and phylogenetic analysis (Figure 4B) with that from other organisms indicated that VdFKBP12 was evolutionarily conserved. To assess the role of *VdFKBP12* gene, knockout transformants (aaa*fkbp12*) were generated by using homologous recombination gene deletion strategy (Supplementary Figure 2). No aberrant phenotype was observed under normal condition (Figure 4C). RAP sensitivity test showed that aaa*fkbp12* mutant resistance to RAP, but the sensitivity to RAP was restored in the complementary strain (aaa*fkbp12* + *FKBP12*) (Figure 4C). Early studies showed that heterologous expressing *ScFKBP12* in *Arabidopsis* can restore the sensitivity to RAP (Sormani et al., 2007; Ren et al., 2012). To further confirm the VdFKBP12 function on bridging the interaction between TOR and RAP, the *VdFKBP12* gene was introduced into *Arabidopsis* and produced *VdFKBP12* overexpression transgenic *Arabidopsis* lines. The transgenic lines displayed the sensitivity to RAP compared with WT *Arabidopsis* (Figure 4D). Results showed that all transgenic lines displayed shorter primary root length, smaller cotyledon and the decreasing fresh weight compared with WT *Arabidopsis* (Figure 4D,E), and this observation was consistent with that *ScFKBP12* overexpression line (BP12-2) in *Arabidopsis* (Ren et al., 2012). These results indicated that RAP inhibits the activity of VdTOR protein by VdFKBP12 in *V. dahliae*.

### Analysis of Gene Expression Profile Under VdTOR Inhibition

**FIGURE 5:**
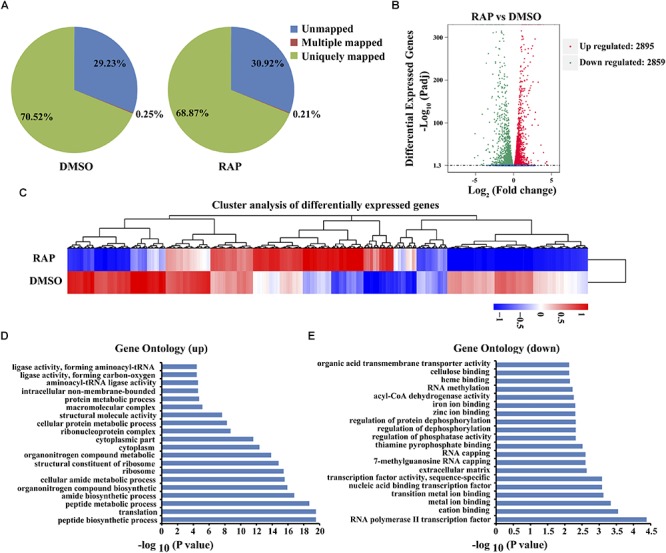
RNA-seq analysis of *V. dahliae* hyphae treated with DMSO and RAP. **(A)** Proportions of clean reads of unmapped, mapped to multiple genes and mapped to unique genes, which were plotted by three replicates of RAP and DMSO. **(B)** The number of down-regulated and up-regulated differentially expressed genes for RAP and DMSO treatment. **(C)** Cluster analysis of differentially expressed genes for RAP and DMSO treatment. **(D)** Significantly up-regulated enriched gene ontology for RAP treatment in the RNA-seq database. Gene ontology was ranked by their significance. **(E)** Significantly down-regulated enriched gene ontology for RAP treatment in the RNA-seq database. Gene ontology was ranked by their significance.

To further elucidate the function of VdTOR signaling pathway on vegetative growth of *V. dahliae*, gene expression profile analysis was performed in *V. dahliae* hyphae under the condition of VdTOR inhibition. RNA-seq was conducted in *V. dahliae* treated with 5 nM RAP and equal volume of DMSO as control, respectively. After stringent quality checking and data cleaning, approximately 70% of the reads can be mapped to the annotated *V. dahliae* genome (Figure 5A). 5754 differentially expressed genes (DEGs) were found between RAP treatment and DMSO control, of which 2,895 DEGs were up-regulated and 2,859 DEGs were down-regulated (Figure 5B,C). Some DEGs were randomly selected from the RNA-seq data to verify the reliability of RNA-seq data by quantitative real-time PCR. The result displayed the same trends as gained in RNA-seq data (Supplementary Figure 3).

To understand the function of these DEGs, GO assignments and enrichments were analyzed. A total of 214 up-regulated GO terms and 139 down-regulated GO terms were enriched (Supplementary Table 3). In the up-regulated GO pathway category, peptide biosynthetic process (GO: 0043043) was the most significant enrichment (Figure 5D). In the down-regulated group, RNA polymerase II transcription factor activity (GO: 0000981) and cation binding (GO: 0043169) were highly represented (Figure 5E). These data showed that VdTOR regulates multiple cellular processes in *V. dahliae*.

### DEGs Involved in the Regulation of Cell Growth in *V. dahliae*

**FIGURE 6:**
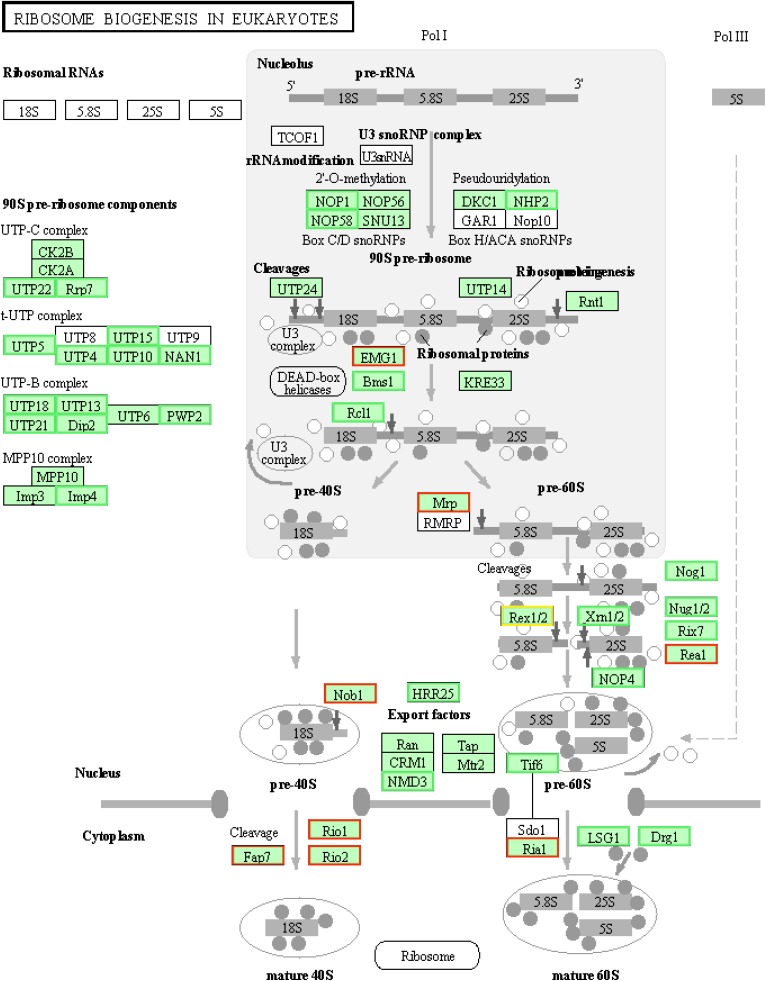
TOR inhibition by RAP induced transcription level changes of ribosome biogenesis-related genes in *V. dahliae*. Expression changes of genes in the ribosome biogenesis in eukaryotes. Green boxes, down-regulated genes. Red boxes, up-regulated genes.

The process of ribosome biogenesis is conserved from prokaryotes to eukaryotes. TORC1 controls the transcription of genes encoding ribosomal proteins and ribosome biogenesis in response to extracellular and intracellular signals in plants, mammals, and yeasts (Wei and Zheng, 2009; Ren et al., 2011; Chauvin et al., 2014; Kos-Braun and Kos, 2017). Through analysis of the RNA-seq data, we found 47 DEGs associated with ribosome biogenesis genes, including 36 down-regulated genes and 11 up-regulated genes, were assigned to the “ribosome biogenesis in eukaryotes” KEGG pathway (Figure 6 and Supplementary Table 4). Within these DEGs, the genes encoding nucleolar proteins 4 and 58 (NOP4 and NOP58) and U3 small nuclear RNA-associated proteins were down-regulated. These ribosomal core proteins combine with small nucleolar RNAs to form small nucleolar ribonucleoproteins that play an indispensible role in ribosome biogenesis (Sun and Woolford, 1994; Gautier et al., 1997; Qiu et al., 2008). These results indicated that VdTOR involved in the regulation of ribosome biogenesis in *V. dahliae*.

Carbohydrate metabolism and synthesis of proteins and lipids are important limiting factors for cells growth and proliferation (Ren et al., 2012; Yuan et al., 2013; Saxton and Sabatini, 2017). Carbon metabolism, amino acids metabolism, and fatty acid metabolism were changed in the RNA-seq data (Table 2). Particularly, the genes encoding rate-limiting enzymes of carbohydrate metabolism and biosynthesis of amino acids such as fructose-bisphosphate aldolase and isocitrate dehydrogenase were down-regulated, but expression levels of several genes involved in nitrogen metabolism such as glutamine synthetase, ammonium permease and NAD-specific glutamate dehydrogenase were up-regulated (Table 2). Additionally, the down-regulation of rate-limiting enzyme fatty acid synthase documented that the suppression of fatty acid biosynthesis of *V. dahliae* by RAP (Table 2). Taken together, the RNA-seq data showed that multiple metabolic processes were affected by RAP, especially carbon metabolism, amino acids and fatty acid metabolism. Metabolic checkpoints of nutrient sensors dictate cell fate in response to metabolic fluctuations (Green et al., 2014), indicating that the disruption of metabolism homeostasis by RAP may contribute to the inhibition of cell growth in *V. dahliae*.

**Table 2:** DEGs of carbon metabolism, amino acids metabolism, and fatty acid metabolism in RNA-seq data.

Gene ID	P-adjusted	Log_2_ (fold change)	Annotation
**Carbon metabolism**			
VDAG_JR2_Chr5g11740	6.55E-243	2.1995	DAK1_SCHPO Dihydroxyacetone kinase 1
VDAG_JR2_Chr6g00530	6.24E-117	1.4242	STDH_YEAST Catabolic L-SERINE/THREONINE DEHYDRATASE
VDAG_JR2_Chr6g05720	1.24E-179	1.1014	CYSD_EMENI *O-acetylhomoserine (thiol)-lyase*
VDAG_JR2_Chr1g16490	8.02E-221	0.88895	TKT_PICST Transketolase
VDAG_JR2_Chr1g06800	3.16E-166	0.8717	FDH_EMENI Probable formate dehydrogenase
VDAG_JR2_Chr8g00100	3.77E-53	0.83258	GNL_ZYMMO Gluconolactonase
VDAG_JR2_Chr1g26020	1.04E-60	0.82823	TAL1_FUSO4 Transaldolase
VDAG_JR2_Chr8g05000	4.53E-178	0.79837	ENO_NEUCR Enolase
VDAG_JR2_Chr7g09140	1.42E-130	0.77503	6PGL_SCHPO Probable 6-phosphogluconolactonase
VDAG_JR2_Chr5g03990	1.24E-132	0.75365	CYSD_EMENI *O-acetylhomoserine(thiol)-lyase*
VDAG_JR2_Chr5g03660	4.5E-13	0.71945	RPIB_COCIM Putative ribose 5-phosphate isomerase
VDAG_JR2_Chr5g03650	3.35E-18	0.61927	DAK_PICAN Dihydroxyacetone kinase
VDAG_JR2_Chr3g09290	2.91E-31	0.61754	SERA1_ARATH D-3-phosphoglycerate dehydrogenase 1
VDAG_JR2_Chr7g02520	2.55E-4	0.56951	S17P_SPIOL Sedoheptulose-1,7-bisphosphatase
VDAG_JR2_Chr7g08500	2.09E-34	0.56335	ESTD_RAT *S-formylglutathione hydrolase*
VDAG_JR2_Chr1g05670	1.48E-27	0.56214	KPYK_HYPJE Pyruvate kinase
VDAG_JR2_Chr3g02770	1.51E-29	0.56089	GNTK_SCHPO Probable gluconokinase
VDAG_JR2_Chr1g18800	5.12E-26	0.50357	MTHR1_SCHPO Methylenetetrahydrofolate reductase 1
VDAG_JR2_Chr2g07820	2.14E-67	-0.55694	MASY_NEUCR Malate synthase, glyoxysomal
VDAG_JR2_Chr3g07200	2.69E-36	-0.59037	GCST_SCHPO Probable aminomethyltransferase
VDAG_JR2_Chr1g27590	7.71E-48	-0.7012	ACEB_HYPAI 2-methylisocitrate lyase
VDAG_JR2_Chr6g03660	1.19E-38	-0.77359	ECHM_DICDI Probable enoyl-CoA hydratase
VDAG_JR2_Chr4g08690	1.19E-76	-0.79738	THIL_DANRE Acetyl-CoA acetyltransferase
VDAG_JR2_Chr1g27600	2.6E-76	-0.85538	PRPC_NECHA 2-methylcitrate synthase
VDAG_JR2_Chr5g04870	5.25E-36	-1.0332	SDHL_BOVIN L-SERINE DEHYDRATASE/L-THREONINE DEAMINASE
VDAG_JR2_Chr1g27120	3.78E-190	-1.1935	GCSP_SCHPO Putative glycine dehydrogenase
VDAG_JR2_Chr3g05350	8.72E-182	-1.5276	MMSA_HUMAN Methylmalonate-semialdehyde dehydrogenase
VDAG_JR2_Chr1g06910	0	-2.9926	MAOX_DICDI NADP-dependent malic enzyme
VDAG_JR2_Chr5g11040	0	-4.1532	ALOX1_PICPG Alcohol oxidase 1
**Amino acids metabolism**			
VDAG_JR2_Chr5g07400	0	2.8585	GLNA_COLGL Glutamine synthetase
VDAG_JR2_Chr8g08530	0	1.7115	CYS3_YEAST Cystathionine gamma-lyase
VDAG_JR2_Chr1g09740	9.02E-63	1.6579	DHE4_BOTFU NADP-specific glutamate dehydrogenase
VDAG_JR2_Chr6g00530	6.24E-117	1.4242	STDH_YEAST Catabolic L-SERINE/THREONINE DEHYDRATASE
VDAG_JR2_Chr7g05140	3.7E-39	1.2732	UAPA_EMENI Uric acid-xanthine permease
VDAG_JR2_Chr6g05720	1.24E-179	1.1014	CYSD_EMENI *O-acetylhomoserine (thiol)-lyase*
VDAG_JR2_Chr1g16490	8.02E-221	0.88895	TKT_PICST Transketolase
VDAG_JR2_Chr1g26020	1.04E-60	0.82823	TAL1_FUSO4 Transaldolase
VDAG_JR2_Chr8g05000	4.53E-178	0.79837	ENO_NEUCR Enolase
VDAG_JR2_Chr1g14730	3.02E-123	0.77108	GLNA_COLGL Glutamine synthetase
VDAG_JR2_Chr5g03990	1.24E-132	0.75365	CYSD_EMENI *O-acetylhomoserine (thiol)-lyase*
VDAG_JR2_Chr5g03660	4.5E-13	0.71945	RPIB_COCIM Putative ribose 5-phosphate isomerase
VDAG_JR2_Chr6g07790	0.04061	0.6344	YOOH_SCHPO Putative xanthine/uracil permease
VDAG_JR2_Chr3g09290	2.91E-31	0.61754	SERA1_ARATH D-3-phosphoglycerate dehydrogenase 1
VDAG_JR2_Chr1g05670	1.48E-27	0.56214	KPYK_HYPJE Pyruvate kinase
VDAG_JR2_Chr3g01780	1.17E-25	0.54709	AROG_YEAST
VDAG_JR2_Chr1g14860	1.5 E-6	-0.30062	Phospho-2-dehydro-3-deoxyheptonate aldolase
VDAG_JR2_Chr4g09540	4.75E-18	-0.53515	IDHP_ASPNG Isocitrate dehydrogenase [NADP], mitochondrial ALF_NEUCR Fructose-bisphosphate aldolase
VDAG_JR2_Chr3g13140	1.83E-6	-0.55234	LEU3_ACRCH 3-isopropylmalate dehydrogenase
VDAG_JR2_Chr3g08840	7.94E-19	-0.85033	BCA1_YEAST Branched chain amino acid aminotransferase
VDAG_JR2_Chr1g27600	2.6E-76	-0.85538	PRPC_NECHA 2-methylcitrate synthase
VDAG_JR2_Chr7g03210	2.02E-17	-0.96402	BCAL2_ARATH Branched chain amino acid aminotransferase protein 2
VDAG_JR2_Chr2g07090	6.64E-140	-1.003	BCA1_SCHPO Branched chain amino acid aminotransferase
VDAG_JR2_Chr5g04870	5.25E-36	-1.0332	SDHL_BOVIN L-SERINE DEHYDRATASE/L-THREONINE DEAMINASE
VDAG_JR2_Chr3g01510	1.51E-62	-1.0716	AATR1_SCHPO Aromatic amino acid aminotransferase
VDAG_JR2_Chr2g03830	0	-2.9695	ARGI_NEUCR Arginase
**Fatty acid metabolism**			
VDAG_JR2_Chr1g20590	6.19E-32	-0.41016	FAS1_YARLI Fatty acid synthase subunit beta
VDAG_JR2_Chr1g20610	2.42E-84	-0.63209	FAS2_PENPA Fatty acid synthase subunit alpha
VDAG_JR2_Chr4g12440	2.22E-12	-0.6338	FAD12_MORAP Delta(12) fatty acid desaturase
VDAG_JR2_Chr1g18160	9.56E-48	-0.63517	LCF1_YEAST Long-chain fatty acid-CoA ligase 1
VDAG_JR2_Chr6g03660	1.19E-38	-0.77359	ECHM_DICDI Probable enoyl-CoA hydratase, mitochondrial
VDAG_JR2_Chr4g08690	1.19E-76	-0.79738	THIL_DANRE Acetyl-CoA acetyltransferase, mitochondrial
VDAG_JR2_Chr4g08190	9.70E-159	-1.0347	ACO1_AJECA Acyl-CoA desaturase


### DEGs Involved in the Regulation of Invasion in *V. dahliae*

Cell wall degrading enzymes produced by phytopathogenic fungi have been proved as virulence factors involving in fungal infection processes (Brito et al., 2006; Mori et al., 2008; Kubicek et al., 2014; Quoc and Chau, 2017), which was also reported in *V. dahliae* (Cooper and Wood, 1980; Tzima et al., 2011). CWDEs including cellulases, hemicellulases and pectinases were changed under VdTOR inhibition by RAP in the RNA-seq data (Supplementary Table 5). Furthermore, 70.27% differentially expressed CWDE genes were down-regulated (Table 3). Importantly, some pivotal genes of CWDEs such as endoglucanases, exoglucanases (cellobiohydrolases), xyloglucanase, xylanase, polygalacturonase and pectate lyase were significantly down-regulated (Table 3), implying that VdTOR plays a role in the regulation of CWDEs. Besides, *VdNLP2* and *VdNLP3*, which encoded NEP1-like proteins of inducing necrotic lesions and triggering defense responses, were down-regulated 1.93- and 1.76-fold, respectively. The gene *VDH1* of encoding a hydrophobin, which played a role in microsclerotia development and the mutant decreased microsclerotia production in *V. dahliae* (Klimes and Dobinson, 2006), was also down-regulated 2.68-fold in RNA-seq data (Supplementary Table 6). These observations implied that VdTOR has a positive role in the regulation of invasion and virulence in *V. dahliae*.

**Table 3:** Representative down-regulated DEGs of CWEDs in transcriptome.

Gene ID	P-adjusted	Log_2_ (fold change)	Annotation
VDAG_JR2_Chr1g28900	1.13E-19	-5.1275	CBHA_ASPFU Probable 1,4-beta-D-glucan cellobiohydrolase A
VDAG_JR2_Chr4g11280	9.95E-76	-5.0736	PLYF_ASPTN Probable pectate lyase F
VDAG_JR2_Chr2g00430	6.09E-33	-3.2231	PLYE_NEOFI Probable pectate lyase E
VDAG_JR2_Chr1g06240	0	-3.0679	BGLF_ASPFU Probable beta-glucosidase F
VDAG_JR2_Chr1g28940	1.74E-07	-2.4926	PLYB_COLGL Pectate lyase B
VDAG_JR2_Chr4g12450	2.26E-181	-2.4638	E13B_CELCE Glucan endo-1,3-beta-glucosidase
VDAG_JR2_Chr1g21910	2.89E-277	-2.3234	CBHB_ASPFU Probable 1,4-beta-D-glucan cellobiohydrolase B
VDAG_JR2_Chr5g09380	9.84E-107	-2.2444	EGLD_ASPFU Probable endo-beta-1,4-glucanase D
VDAG_JR2_Chr3g12300	6.49E-17	-2.1704	PLYB_EMENI Pectate lyase plyB
VDAG_JR2_Chr4g11890	5.21E-67	-1.843	GUX6_HUMIN Exoglucanase-6A
VDAG_JR2_Chr3g09940	2.96E-31	-1.6013	ENG2_SCHPO Putative endo-1,3(4)-beta-glucanase 2
VDAG_JR2_Chr6g09790	2.07E-06	-1.5294	EGLD_ASPOR Probable endo-beta-1,4-glucanase D
VDAG_JR2_Chr4g09870	0.01194	-1.4107	EGLD_NEOFI Probable endo-beta-1,4-glucanase D
VDAG_JR2_Chr4g01600	2.89E-08	-1.3872	PGLRX_ASPFU Probable exopolygalacturonase X
VDAG_JR2_Chr2g00660	7.46E-23	-1.1696	GUX1A_NEUCR Exoglucanase 1
VDAG_JR2_Chr1g18750	0.00551	-1.0915	Y584_MYCTU Uncharacterized glycosidase Rv0584
VDAG_JR2_Chr4g11100	1.56E-14	-1.0353	MANC_EMENI Mannan endo-1,4-beta-mannosidase C
VDAG_JR2_Chr2g04070	6.57E-07	-1.0335	XYLO_PRERU Putative beta-xylosidase
VDAG_JR2_Chr8g11250	7.80E-08	-1.0267	PLYD_EMENI Probable pectate lyase D
VDAG_JR2_Chr3g13470	2.37E-14	-1.0172	XYN1_MAGO7 Endo-1,4-beta-xylanase 1
VDAG_JR2_Chr7g03190	0.000388	-0.9875	EGLD_NEOFI Probable endo-beta-1,4-glucanase D
VDAG_JR2_Chr2g02490	1.38E-08	-0.9245	AGALB_ASPFU Probable alpha-galactosidase B
VDAG_JR2_Chr4g08080	4.99E-21	-0.9106	EGLX_ASPFU Probable endo-1,3(4)-beta-glucanase
VDAG_JR2_Chr4g11060	0.000179	-0.9039	CE12C_MAGO7 Endoglucanase cel12C
VDAG_JR2_Chr1g28970	5.07E-08	-0.8981	AGALD_EMENI Alpha-galactosidase D
VDAG_JR2_Chr4g02950	2.85E-10	-0.8671	MANC_ASPTN Probable mannan endo-1,4-beta-mannosidase C
VDAG_JR2_Chr4g10880	2.07E-06	-0.85316	PGLR2_JUNAS Polygalacturonase
VDAG_JR2_Chr2g04540	1.64E-24	-0.7861	MANA_RHOM4 Mannan endo-1,4-beta-mannosidase
VDAG_JR2_Chr7g01060	1.89E-53	-0.7811	MANBB_THIHE Beta-mannosidase B
VDAG_JR2_Chr1g28360	5.30E-09	-0.7564	PLYC_ASPFU Probable pectate lyase C
VDAG_JR2_Chr3g12920	1.07E-09	-0.7449	XG74_HYPJQ Xyloglucanase
VDAG_JR2_Chr6g00450	2.30E-94	-0.742	EXG1_COCCA Glucan 1,3-beta-glucosidase
VDAG_JR2_Chr4g09790	1.33E-107	-0.7227	CE12C_MAGO7 Endoglucanase cel12C
VDAG_JR2_Chr1g06450	5.47E-20	-0.6796	DCW1_ASHGO Mannan endo-1,6-alpha-mannosidase DCW1
VDAG_JR2_Chr5g03940	8.91E-12	-0.6735	XYNB_BACPU Beta-xylosidase
VDAG_JR2_Chr7g03350	3.09E-19	-0.6291	RGXB_ASPNC Alpha-L-rhamnosidase rgxB
VDAG_JR2_Chr6g06610	7.73E-06	-0.6092	CEL6B_PODAN 1,4-beta-D-glucan cellobiohydrolase CEL6B
VDAG_JR2_Chr8g09390	0.005378	-0.5962	XYN1_HUMGT Endo-1,4-beta-xylanase 1
VDAG_JR2_Chr8g05830	0.000267	-0.5553	EXG1_COCCA Glucan 1,3-beta-glucosidase
VDAG_JR2_Chr2g03150	1.27E-08	-0.549	CE12B_MAGO7 Endoglucanase cel12B
VDAG_JR2_Chr1g15250	7.21E-07	-0.5353	BGLM_ASPFN Probable beta-glucosidase M


**FIGURE 7:**
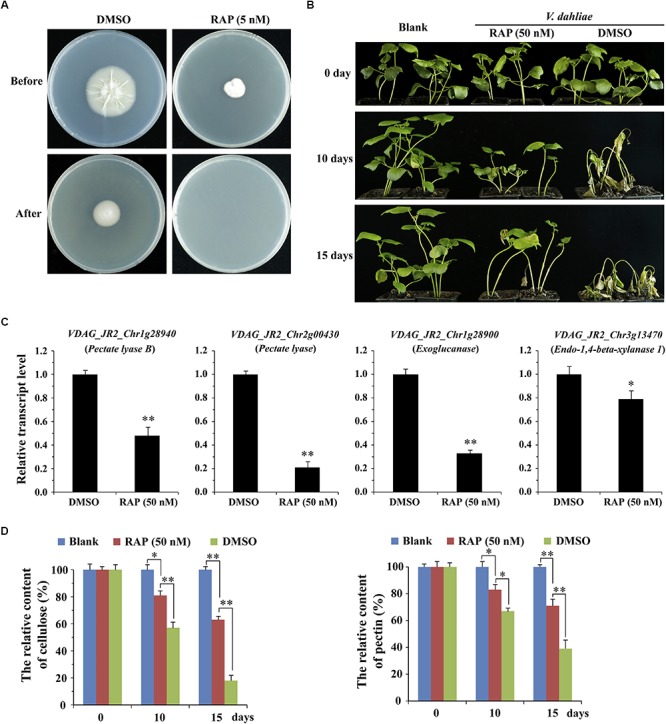
RAP attenuates the invasive ability of *V. dahliae*. **(A)** Effect of RAP on penetration of cellophane membranes by *V. dahliae*. Fungal colonies were grown for 7 days at 27°C on top of cellophane membranes on plates containing RAP (5 nM) (Before). The cellophane with the fungal colony was removed and plates were incubated for 3 days to determine the presence of mycelial growth on the plate (After). **(B)** Phenotype of cotton plants (*Gossypium hirsutum*) at 0, 10, and 15 days post-inoculation with conidia suspension supplemented with or without 50 nM RAP. **(C)** The relative transcript levels of genes associated with CWDEs. The data represents the mean ± SD of *n* = 3 independent experiments. Asterisks denote Student’s *t*-test significant difference compared with DMSO (^∗^*P* < 0.05; ^∗∗^*P* < 0.01). **(D)** The relative content of cellulose and pectin of cotton roots (*Gossypium hirsutum*) at 0, 10, and 15 days post-inoculation with conidia suspension supplemented with or without 50 nM RAP. The data represents the mean ± SD of *n* = 3 independent experiments. Asterisks denote Student’s *t*-test significant difference compared with RAP (^∗^*P* < 0.05; ^∗∗^*P* < 0.01).

To further determine the effect of VdTOR on invasion in *V. dahliae*, cellophane penetration assay was performed to verify invasive growth of *V. dahliae*. The *V. dahliae* wild-type strain was incubated on top of cellophane membrane on PDA medium supplemented with RAP. The hyphae of *V. dahliae* can efficiently penetrate cellophane membrane on PDA medium, but not PDA medium containing RAP, suggesting an inhibitory effect of *V. dahliae* on cellophane penetration by RAP treatment (Figure 7A). Importantly, the main component of cellophane is cellulose, which mimics the component of plant cell wall, implying that RAP weakens the activity of CWDEs by inhibiting VdTOR activity. Cotton (*Gossypium hirsutum*) plants were also used to test the pathogenicity of *V. dahliae* treated with RAP. After inoculation for 10 days, cotton plants that infection with conidia suspension of *V. dahliae* occurred wilting of leaves. However, the disease severity of cotton plants that were infected with the conidia suspension containing RAP was reduced compared to that observed with the conidia suspension containing DMSO (Figure 7B). To confirm that the reduction in pathogenicity was caused by reducing the activity of CWDEs, we examined the transcription levels of genes associated with CWDEs. These genes including pectate lyase (*VDAG_JR2_Chr1g28940* and *VDAG_JR2_Chr2g00430*), exoglucanase (*VDAG_JR2_Chr1g28900*) and endo-1,4-beta-xylanase 1 (*VDAG_JR2_Chr3g13470*) were significantly down-regulated in TOR-inhibited cells by RAP (Figure 7C). Meanwhile, we also measured the content of cellulose and pectin. The content of cellulose and pectin were significantly increased in cotton roots infected with the conidia suspension-containing RAP compared with the plants that were infected by conidia suspension of *V. dahliae* (Figure 7D). Taken together, these results suggested that VdTOR positively regulates the pathogenicity of *V. dahliae*.

## Discussion

*V. dahliae* is a phytopathogenic fungus that causes serious wilt disease in various plants including many economically important crops, especially for cotton (Agrios, 2005; Chen et al., 2016, 2017). TOR signaling pathway controls a wide variety of cellular processes in response to nutrients, growth factors, stresses and other environmental signals (Yuan et al., 2013; Rexin et al., 2015; Dobrenel et al., 2016; Saxton and Sabatini, 2017). Analyze the functions of VdTOR signaling pathway, which is important to gain insight into molecular processes involved in the cell growth and pathogenicity of *V. dahliae*. In this study, we provided some insights into how VdTOR modulates cell growth and pathogenicity through controls multiple cellular processes. We identified the putative components of VdTOR signaling pathway including TORC1 and TORC2 based on released genome database of *V. dahliae* (Table 1). The high similarity of the kinase domain of TOR protein was obtained among *V. dahliae* and other organisms (Figure 3), suggesting TOR is a structurally conserved protein in various species.

Rapamycin is an allosteric inhibitor of TOR and was approved as an immunosuppressant (Zaragoza et al., 1998). Since the function mutation of plants FKBP12 results in plants insensitivity to RAP (Sormani et al., 2007; Ren et al., 2012), RAP can be used as a potential fungicide for plant fungal diseases. For example, in comparison with chemical fungicides tebuconazole and carbendazim, RAP showed a stronger inhibitory effect on hyphal growth of *Fusarium graminearum* (Yu et al., 2014). Owing to its specificity, RAP has proven to be an invaluable drug in the discovery of TOR and as a pharmacological tool to dissect TOR’s cellular function (Benjamin et al., 2011; Ren et al., 2012). RAP was applied to elucidate the function of VdTOR in *V. dahliae*. As expected, RAP can effectively inhibit mycelial growth and conidial development of *V. dahliae* in a dose dependent manner (Figure 1). To further confirm whether RAP mediates the inhibition of VdTOR protein by VdFKBP12, aaa*vdfkbp12* mutant and *VdFKBP12* overexpression transgenic *Arabidopsis* lines were generated. RAP sensitivity test showed that aaa*vdfkbp12* mutant resistance to RAP, but *VdFKBP12* overexpression transgenic *Arabidopsis* lines were sensitive to RAP (Figure 4), suggesting that the ternary complex of RAP-VdFKBP12-FRB domain of VdTOR is necessary for TOR inhibition.

Due to the high specificity and minimal off-target effects of RAP, it was employed to further elucidate the function of VdTOR signaling pathway by RNA sequencing. The RNA-seq analysis showed that VdTOR inhibition resulted in changes in many metabolic processes (Figure 5, 6 and Table 2). Importantly, the disruption of carbon metabolism, biosynthesis of proteins and fatty acid metabolism destroyed metabolic homeostasis. Analysis of the RNA-seq data suggested that the inhibitory effect of RAP on cell growth of *V. dahliae* is most likely due to disruption the homeostasis of some important metabolic processes. The TOR kinase is a central regulator of growth and metabolism in all eukaryotic species including animals, plants and fungi (Yang et al., 2013; Rexin et al., 2015; Saxton and Sabatini, 2017). Metabolism changes from anabolism to catabolism leads to a massive accumulation of starch, triacylglycerols and amino acids after TOR inhibition (Imamura et al., 2015; Juppner et al., 2018). Interestingly, TOR inhibition increased nitrogen uptake and activities of glutamine synthetase and glutamine oxoglutarate aminotransferase in Chlamydomonas (Mubeen and Juppner, 2018). We observed that VdTOR inhibition led to transcriptional up-regulation of genes involved in nitrogen metabolism such as glutamine synthetase, the main nitrogen assimilating enzymes. This result is, at least in part, in agreement with previous study showing that RAP activates expression of nitrogen metabolism related genes in *Chlamydomonas reinhardtii* and *Fusarium fujikuroi* (Teichert et al., 2006; Mubeen and Juppner, 2018). Furthermore, RAP also altered the expression of important genes associated with CWDEs and virulence (Figure 7, Table 3, and Supplementary Table 5). In the process of recognition of plant pathogenic fungi and the host, CWDEs secreted by phytopathogenic fungi can degrade the cell wall of the host plant, which is conducive to the invasion and colonization of pathogenic fungi (Quoc and Chau, 2017; Liu et al., 2018). Transcriptional regulation of genes encoding CWDEs was controlled by transcription factors. The zinc finger transcription factor XlnR is a major activator of CWDEs in pathogenic fungi. Deletion of *XlnR* gene lacked transcriptional activation of xylanase and cellulase genes which resulted in failure in xylan and cellulose degradation (Calero-Nieto et al., 2007; Battaglia et al., 2011; Klaubauf et al., 2014). Besides, other transcription factors such as ACEII, PacC and CRE were documented to be involved in regulating the expression of pectinases, cellulases and xylanases encoding genes (Aro et al., 2001, 2005; Quoc and Chau, 2017). TOR and the bZIP protein MeaB control vegetative hyphal invasion and root adhesion in plant pathogenic fungi (Lopez-Berges et al., 2010). These observations implied that transcription factors play important roles in various intracellular processes regulated by TOR signaling pathway. Whether TOR regulates the activity of CWDEs through some transcription factors such as zinc finger proteins and bZIP proteins still needs further study.

## Conclusion

In conclusion, TOR specific inhibitor RAP can inhibit the mycelial growth of *V. dahliae* in a dose dependent manner, suggesting that VdTOR plays an essential role in hyphal growth and development. These observations indicated direct inhibitory effects of RAP on the hyphal growth of *V. dahliae* and provided some insights into the interaction between RAP and plant pathogens. RNA-seq analysis indicated that VdTOR inhibition resulted in changes in various metabolic processes. Importantly, many genes of CWDEs were down-regulated during VdTOR inhibition by RAP, suggesting that VdTOR positively involved in the regulation of CWDEs. Further infection assay showed that the pathogenicity of *V. dahliae* and occurrence of Verticillium wilt can be blocked by RAP, indicating that RAP can be used as a potential bio-fungicide instead of chemical fungicides to prevent the occurrence of Verticillium wilt.

## Data Availability

All datasets analyzed for this study are included in the manuscript and the Supplementary Files.

## Author Contributions

MR, FL, and LL designed the experiments. LL, TZ, YS, XL, LF, and FZ performed the experiments. LL, TZ, and YS analyzed the data. MR and LL wrote the manuscript.

## Conflict of Interest Statement

The authors declare that the research was conducted in the absence of any commercial or financial relationships that could be construed as a potential conflict of interest.

## Footnotes

1 http://fungi.ensembl.org/Verticillium_dahliaejr2/Info/Index
