# Supplementary material for: Functional Characterization of Target of Rapamycin Signaling in Verticillium dahliae
Source: Front Microbiol. 2019 Mar 13;10:501. doi: 10.3389/fmicb.2019.00501 (PMC6424901; doi:10.3389/fmicb.2019.00501)

***Supplementary Material***

**Functional characterization of Target of Rapamycin signaling in *Verticillium dahliae***

**Linxuan Li^1^, Tingting Zhu^1^, Yun Song^2,3^, Xiumei Luo^1^, Li Feng^1^, Fengping Zhuo^1,4^, Fuguang Li^2,3^ and Maozhi Ren^1^**^*^

1 School of Life Sciences, Chongqing University, Chongqing, China

2 Zhengzhou Research Base，State Key Laboratory of Cotton Biology, Zhengzhou University, Zhengzhou 450000, China

3 Institute of Cotton Research, Chinese Academy of Agricultural Sciences, Anyang 455000, China

4 School of Chemistry and Chemical Engineering, Chongqing University of Science and Technology, Chongqing, China.

*Correspondence:

Maozhi Ren
School of Life Sciences
Chongqing University
174 Shazheng ST, Shapingba,
Chongqing, China, 400045
Phone: 86-13527313471
E-mail: renmaozhi@cqu.edu.cn


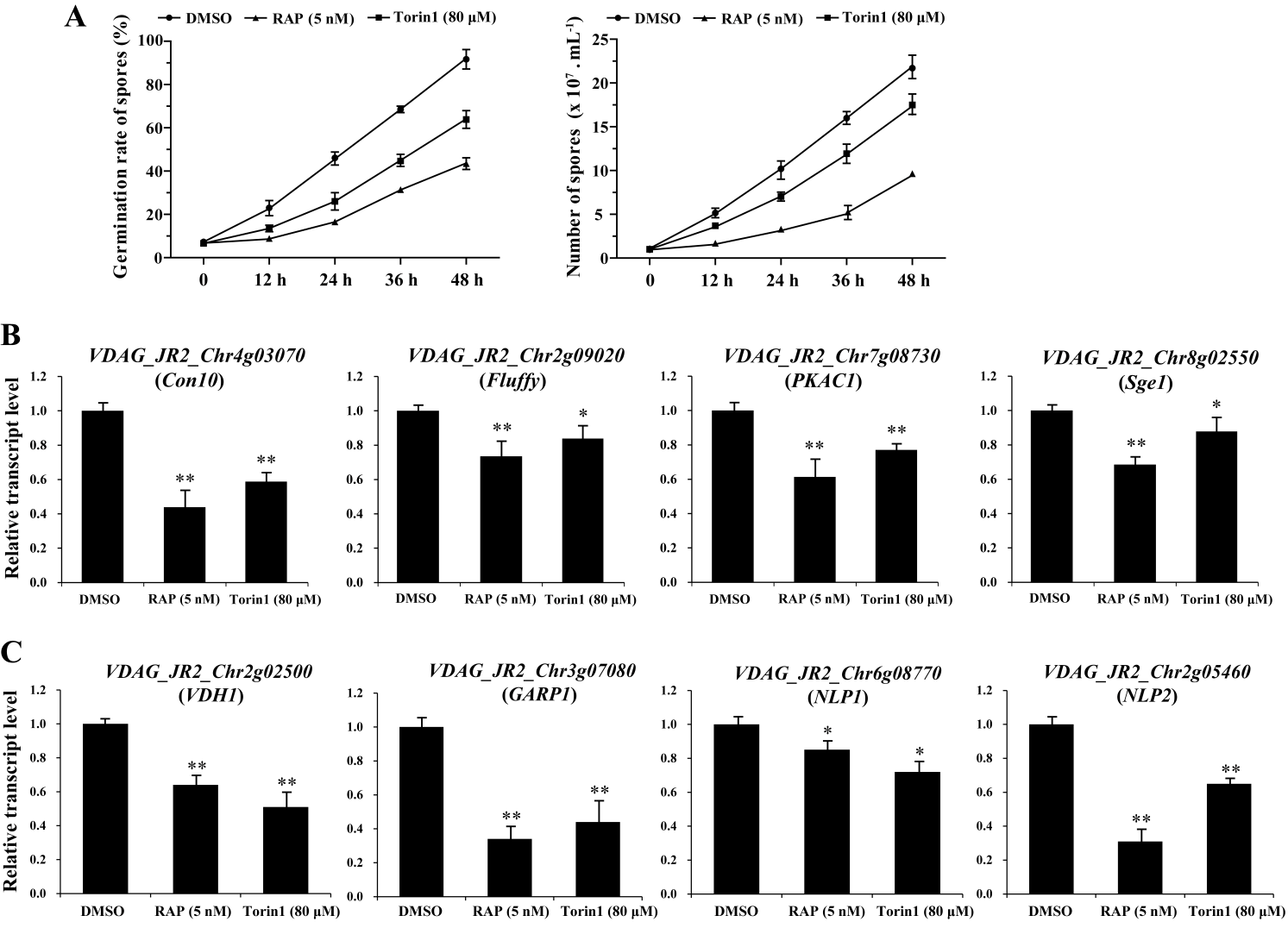


**Supplemental Figure 1｜RAP and Torin1 can inhibit conidia development, vegetative growth and virulence.**

**(A)** Germination of conidia and spore production, hyphae were incubated in PDB medium for 5 days and then treated with RAP and Torin1 from 0 to 48 h. The data represents the mean ± SD of n = 3 independent experiments.

**(B)** Relative expression level of sporulation and germination related genes including *VDAG_JR2_Chr4g03070* (*Vdcon10*), *VDAG_JR2_Chr2g09020* (*Vdfluffy*), *VDAG_JR2_Chr7g08730* (*VdPKAC1*) and *VDAG_JR2_Chr8g02550* (*VdSge1*). Hyphae were incubated with RAP and Torin1 for 24 h. The data represents the mean ± SD of n = 3 independent experiments. Asterisks denote Student’s t test signiﬁcant difference compared with DMSO (*P < 0.05; **P < 0.01).

**(C)** Relative expression level of vegetative growth and virulence related genes including *VDAG_JR2_Chr2g02500* (*VDH1*), *VDAG_JR2_Chr3g07080* (*GARP1*), *VDAG_JR2_Chr6g08770* (*NLP1*) and *VDAG_JR2_Chr2g05460* (*NLP2*). Hyphae were incubated with RAP and Torin1 for 24 h. The data represents the mean ± SD of n = 3 independent experiments. Asterisks denote Student’s t test signiﬁcant difference compared with DMSO (*P < 0.05; **P < 0.01).


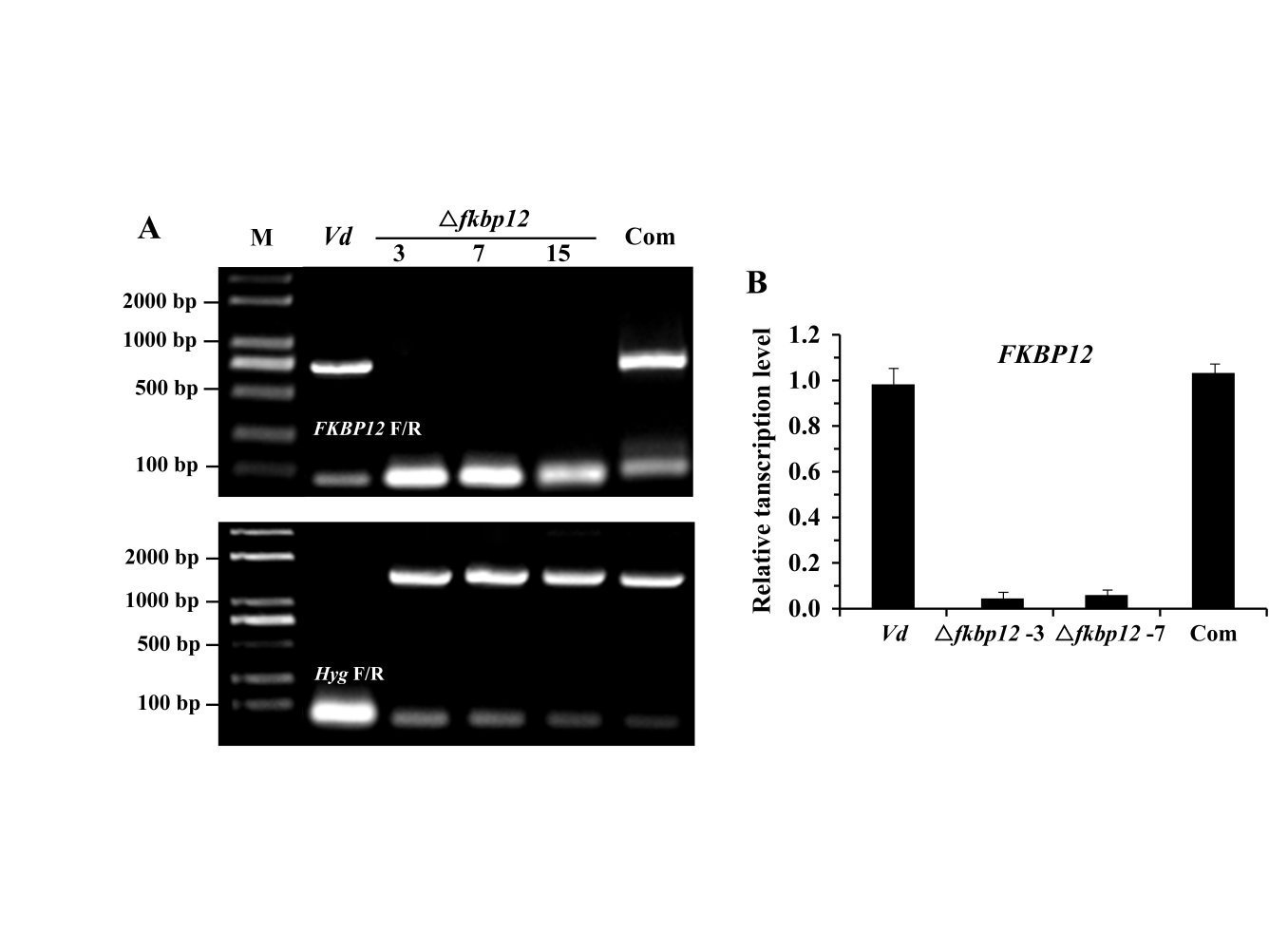


**Supplemental Figure 2｜Verification of △*fkbp12*.**

**(A)** Gel electrophoresis of *FKBP12* gene and *Hyg* cassette. *VdFKBP12* gene and *Hyg* cassette were amplified from *Vd*, △*fkbp12* mutants and Com with *FKBP12* F/R and *Hyg* F/R primers, individually. M, DNA marker, *Vd*, *Verticillium dahliae*; Com, complemented strain.

**(B)** qRT-PCR analysis of *FKBP12* gene expression in the *Vd*, △*fkbp12* mutants and Com. The data represents the mean ± SD of n = 3 independent experiments.


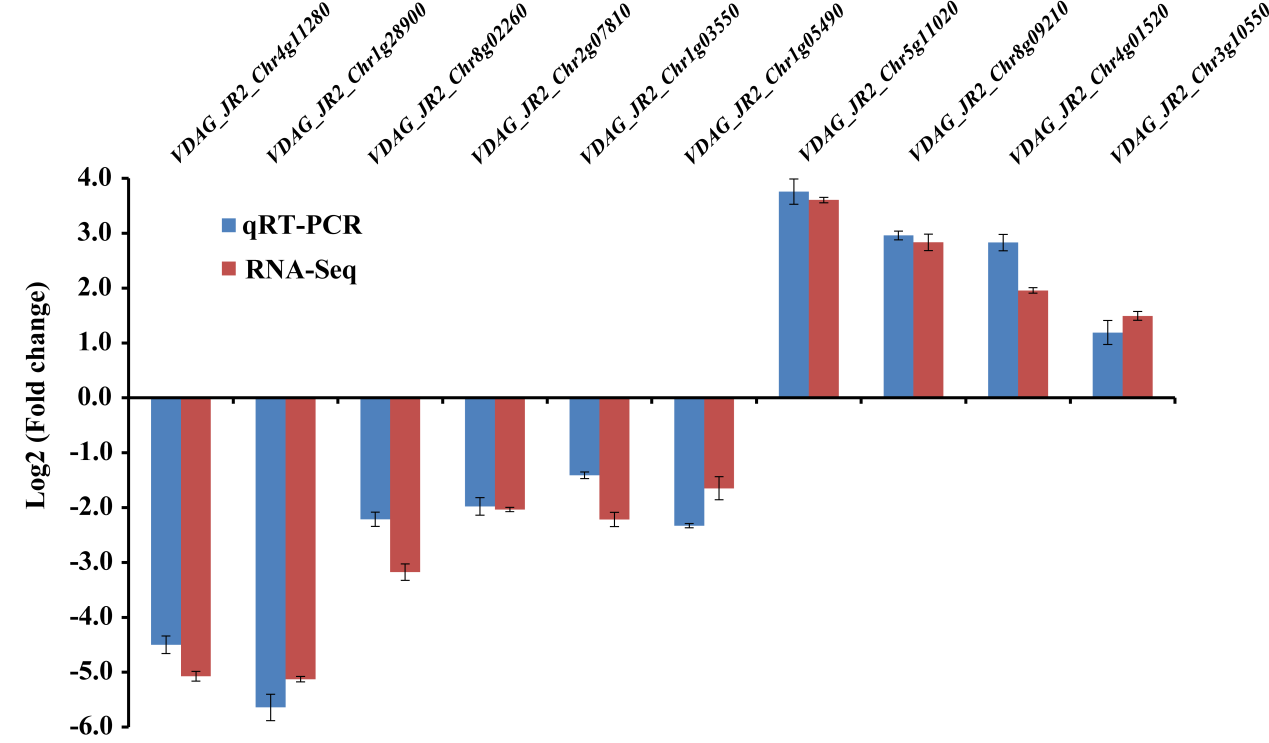


**Supplemental Figure 3｜**Real-time PCR verification of the differentially expressed genes in transcriptome. The data represents the mean ± SD of n = 3 independent experiments.

**Supplementary Table 1｜**Primers used for genes cloning and construction of replacement cassette in this study.

| Primer name | Primer sequence (5’-3’) |
| --- | --- |
| Deletion primers | |
| VdFKBP12 5’-U F | GGTACCAACGCACGACAGCCCGCTTT |
| VdFKBP12 5’-U R | TTCAGGCTTTTTCATGGTGGCCGCGAAAAC |
| VdFKBP12-Hph-F | GTTTTCGCGGCCACCATGAAAAAGCCTGAAC |
| VdFKBP12-Hph-R | ATCTCCACGTCTGATCTATTCCTTTGCCCT |
| VdFKBP12 3’-D F | AGGGCAAAGGAATAGATCAGACGTGGAGAT |
| VdFKBP12 3’-D R | TTAATTAACTGCTGAGCCTCCTTTGACAGC |
| Cloning primers | |
| PVdFKBP12 F | GCGATCGCGGCGAGATGATTGTCGGAGGTC |
| PVdFKBP12 R | GCGGCCGCGGTGGCCGCGAAAACGCGGAGC |
| VdFKBP12 F | GCGGCCGCATGGGCGTCAACGTTATCAC |
| VdFKBP12 R | CCTGCAGGCTGGACCTTCTTCAGCTCGAC |

**Supplementary Table 2｜**Primers used for qRT-PCR

| Primer name | Primer sequence (5’-3’) |
| --- | --- |
| RT *Vd18S rRNA* F | ATTGTTGCTTCGGCGGCTCGTT |
| RT *Vd18S rRNA* R | GCGTTTCGCTGCGTTCTTCATC |
| RT *VdPKAC1* F | CCCTCACCGATTTCGACCTG |
| RT *VdPKAC1* R | CTCGGCGGCATAAAACTTGG |
| RT *VdSge1* F | GTCTGCGGCTAATGTCCC |
| RT *VdSge1* R | GGTCCCATTCATCCCTGT |
| RT *VDH1* F | CTATTGCGATTGCTCTG |
| RT *VDH1* R | GAGCTCAAGGTTTTCGTG |
| RT *VdGARP1* F | ATGCCGCCCAAAAAGCCCTCACCCG |
| RT *VdGARP1* R | TTAATCACTGTCATTGCCATCCAGC |
| RT *VdNLP1* F | CCTCTGCTTCACATTGCC |
| RT *VdNLP1* R | GCCCTTGCTGGTTTCCTT |
| RT *VdNLP2* F | CGCCAACGCCATCCTGTA |
| RT *VdNLP2* R | TCCGCCGTGAAGACCATC |
| RT *VdFKBP12* F | ACTTCGCAACCCGACAAC |
| RT *VdFKBP12* R | CCAGCCACGGATGACTTG |
| RT *VDAG_JR2_Chr4g03070* F | GCATGGACGCCGATAAGCAG |
| RT *VDAG_JR2_Chr4g03070* R | TCGAAAGAGCCCGATGACGC |
| RT *VDAG_JR2_Chr2g09020* F | ATCTTTTGGTCGGTCATCTGGA |
| RT *VDAG_JR2_Chr2g09020* R | GCAGTCAATGGGCTTTTGTTAG |
| RT *VDAG_JR2_Chr2g00430* F | AGTTCTCCCTCGCCATCACC |
| RT *VDAG_JR2_Chr2g00430* R | ACTCCTTGTTGCCGAAGTCC |
| RT *VDAG_JR2_Chr1g28940* F | AAGACAGCCATTGCTAACG |
| RT *VDAG_JR2_Chr1g28940* R | ACCGACACCGATGACACTC |
| RT *VDAG_JR2_Chr1g28900* F | GATGGTGCCATTGAGTGCG |
| RT *VDAG_JR2_Chr1g28900* R | GGTTGCCGAGACGGTAAGG |
| RT *VDAG_JR2_Chr3g13470* F | TACTCCGCTTCATGGCAACC |
| RT *VDAG_JR2_Chr3g13470* R | ACTCCACCAGAGGCGACCTT |

**Supplementary Table 4｜**Representative DEGs of ribosome biogenesis in eukaryotes

| Gene ID | P-adjusted | Log_2_(Fold change) | Annotation |
| --- | --- | --- | --- |

| VDAG_JR2_Chr1g04630 | 1.06E-25 | - 0.57637 | NOG2_NEUCR Nucleolar GTP-binding protein 2 |
| --- | --- | --- | --- |
| VDAG_JR2_Chr6g02800 | 1.13E-18 | -0.55512 | HHP1_SCHPO Casein kinase I homolog hhp1 |
| VDAG_JR2_Chr3g05490 | 7.76E-12 | -0.35339 | ERB1_CHAGB Ribosome biogenesis protein ERB1 RB1 PE=3 |
| VDAG_JR2_Chr4g08440 | 9.17E-12 | -0.25061 | NOG1_ASHGO Nucleolar GTP-binding protein 1 |
| VDAG_JR2_Chr1g06980 | 4.8E-10 | -0.42019 | UTP13_SCHPO U3 small nucleolar RNA-associated protein 13 |
| VDAG_JR2_Chr3g05010 | 5.5E-10 | -0.55275 | UTP18_SCHPO U3 small nucleolar RNA-associated protein 18 |
| VDAG_JR2_Chr1g21640 | 7.77E-09 | -0.32378 | RL1DB_SCHPO Putative ribosome biogenesis protein C8F11 |
| VDAG_JR2_Chr7g07350 | 2.81E-08 | 0.24821 | YF66_SCHPO UPF0665 family protein C23C4 |
| VDAG_JR2_Chr5g00140 | 4.52E-08 | 0.3912 | NOB1_SCHPO 20S-pre-rRNA D-site endonuclease nob1 |
| VDAG_JR2_Chr1g03450 | 1.12E-07 | -0.3266 | UTP17_SCHPO U3 small nucleolar RNA-associated protein 17 |
| VDAG_JR2_Chr8g02660 | 1.19E-07 | -0.21607 | NMD3_YEAST 60S ribosomal export protein NMD3 |
| VDAG_JR2_Chr3g03790 | 2.28E-07 | -0.29188 | YB1C_SCHPO Uncharacterized WD repeat-containing protein C3D6 |
| VDAG_JR2_Chr2g05910 | 4.98E-07 | 0.2265 | TSR1_SCHPO Ribosome biogenesis protein tsr1 |
| VDAG_JR2_Chr1g08410 | 5.61E-07 | -0.31172 | PWP2_NEUCR Periodic tryptophan protein 2 homolog |
| VDAG_JR2_Chr6g04460 | 6.92E-07 | -0.31966 | UTP5_SCHPO U3 small nucleolar RNA-associated protein 5 |
| VDAG_JR2_Chr8g08770 | 7.96E-07 | 0.57533 | ORN_BOVIN Oligoribonuclease, mitochondrial |
| VDAG_JR2_Chr7g07030 | 1.56E-06 | -0.26091 | NOP4_YEAST Nucleolar protein 4 |
| VDAG_JR2_Chr1g02540 | 1.8E-06 | -0.39042 | YB7A_SCHPO Uncharacterized AAA domain-containing protein |
| VDAG_JR2_Chr3g04760 | 2.11E-06 | -0.37751 | RCL1_YEAST RNA 3&apos;-terminal phosphate cyclase-like protein |
| VDAG_JR2_Chr8g04270 | 5.15E-06 | -0.27014 | UTP21_SCHPO U3 small nucleolar RNA-associated protein 21 |
| VDAG_JR2_Chr1g17990 | 8.25E-06 | -0.20922 | BMS1_SCHPO Ribosome biogenesis protein bms1 |
| VDAG_JR2_Chr2g06010 | 9.08E-06 | -0.28963 | AFG2_YEAST ATPase family gene 2 protein |
| VDAG_JR2_Chr1g17140 | 2.28E-05 | 0.19957 | POPI_SCHPO Ribonucleases P/MRP protein subunit pop1 |
| VDAG_JR2_Chr2g01130 | 3.91E-05 | -0.28134 | UTP22_SCHPO U3 small nucleolar RNA-associated protein |
| VDAG_JR2_Chr3g10670 | 6.11E-05 | -0.24592 | NOL10_YEAST Ribosome biogenesis protein ENP2 |
| VDAG_JR2_Chr3g07120 | 7.26E-05 | -0.28766 | NUG1_YEAST Nuclear GTP-binding protein NUG1 |
| VDAG_JR2_Chr4g04900 | 9.06E-05 | -0.14276 | NOP58_ASPCL Nucleolar protein 58 |
| VDAG_JR2_Chr6g00280 | 0.000111 | 0.19601 | RIO1_SCHPO Serine/threonine-protein kinase rio1 |
| VDAG_JR2_Chr1g10830 | 0.000176 | -0.34382 | YTM1_MAGO7 Ribosome biogenesis protein YTM1 |
| VDAG_JR2_Chr7g07840 | 0.000193 | -0.22647 | IF6_VERA1 Eukaryotic translation initiation factor 6 |
| VDAG_JR2_Chr7g02590 | 0.000333 | -0.2397 | UTP4_SCHPO U3 small nucleolar RNA-associated protein 4 |
| VDAG_JR2_Chr2g01650 | 0.00135 | 0.1412 | NEP1_YEAST Ribosomal RNA small subunit methyltransferase |
| VDAG_JR2_Chr8g03740 | 0.001887 | 0.19466 | RIO2_SCHPO Serine/threonine-protein kinase rio2 |
| VDAG_JR2_Chr1g11320 | 0.002013 | -0.16127 | LSG1_YEAST Large subunit GTPase 1 |
| VDAG_JR2_Chr1g28470 | 0.002142 | -0.23555 | NLE1_CHATD Ribosome assembly protein 4 |
| VDAG_JR2_Chr1g14910 | 0.003239 | -0.24098 | UTP15_SCHPO U3 small nucleolar RNA-associated protein 15 |
| VDAG_JR2_Chr1g16780 | 0.003938 | -0.19133 | IMP4_CANGA U3 small nucleolar ribonucleoprotein protein IMP4 |
| VDAG_JR2_Chr1g13090 | 0.009582 | -0.24036 | RRP7_SCHPO Ribosomal RNA-processing protein 7 |
| VDAG_JR2_Chr5g06110 | 0.01351 | 0.12863 | RIA1_SCHPO Ribosome assembly protein 1 |
| VDAG_JR2_Chr1g02320 | 0.013577 | -0.19718 | BRX1_YEAST Ribosome biogenesis protein BRX1 |
| VDAG_JR2_Chr8g08390 | 0.018167 | -0.16918 | YFE9_SCHPO Uncharacterized exonuclease C637 |
| VDAG_JR2_Chr7g07330 | 0.018554 | -0.16281 | UTP10_MAGO7 U3 small nucleolar RNA-associated protein 10 |
| VDAG_JR2_Chr6g07160 | 0.018581 | -0.14671 | XRN2_GIBZE 5&apos;-3&apos; exoribonuclease 2 |
| VDAG_JR2_Chr1g16150 | 0.035428 | -0.10349 | NHP2_YEAST H/ACA ribonucleoprotein complex subunit 2 |
| VDAG_JR2_Chr1g09390 | 0.037027 | 0.18851 | KAD6_RABIT Adenylate kinase isoenzyme 6 |
| VDAG_JR2_Chr2g04750 | 0.044602 | 0.13807 | REXO3_CANAL RNA exonuclease 3 |
| VDAG_JR2_Chr6g07720 | 0.044817 | -0.13474 | FBRL_NEUCR rRNA 2&apos;-O-methyltransferase fibrillarin |


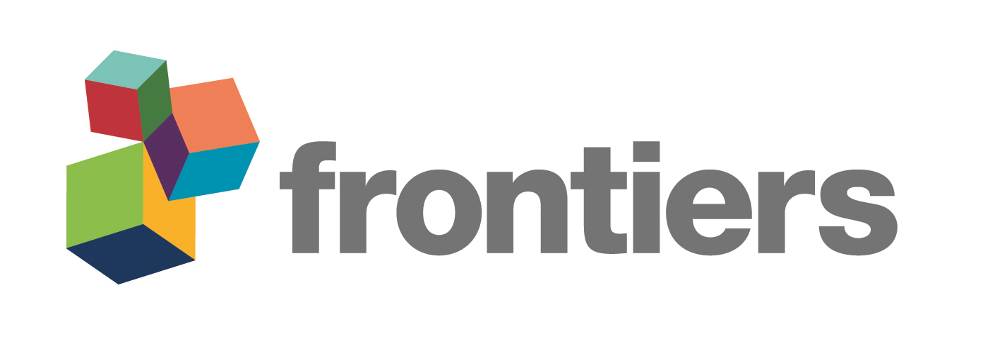

Supplement: Supplementary file 1 [file Data_Sheet_1.docx]
